# Effect of ultrafiltration during hemodialysis on hepatic and total-body water: an observational study

**DOI:** 10.1186/s12882-018-1150-8

**Published:** 2018-12-12

**Authors:** Claire J. Grant, Trevor P. Wade, Charles A. McKenzie, Guido Filler, Christopher W. McIntyre, Shih-Han S. Huang

**Affiliations:** 1 The Lilibeth Caberto Kidney Clinical Research Unit, Victoria Hospital, Western University, A2-344, 800 Commissioners Road East, London, ON N6A 5W9 Canada; 2 Department of Medical Biophysics, Western University, London, ON Canada; 3 Department of Paediatrics, Western University, London, ON Canada; 4 Lawson Health Research, London Health Sciences Centre, London, ON Canada; 5 Division of Nephrology, Department of Medicine, London Health Sciences Centre, London, ON Canada; 6 Department of Pathology & Laboratory Medicine, Western University, London, ON Canada

**Keywords:** Chronic hemodialysis, Ultrafiltration, Intradilyatic hypotension, Fluid overload, Magnetic resonance imaging

## Abstract

**Background:**

The hepatic circulation is involved in adaptive systemic responses to circulatory stress. However, it is vulnerable to both chronic hypervolemia and cardiac dysfunction. The influence of hemodialysis (HD) and ultrafiltration (UF) upon liver water content has been understudied. We conducted a detailed pilot study to characterize the effects of HD upon liver water content and stiffness, referenced to peripheral fluid mobilization and total body water.

**Methods:**

We studied 14 established HD patients without liver disease. Magnetic resonance imaging (MRI) together with ultrasound-based elastography and bioimpedance assessment were employed to measure hepatic water content and stiffness, body composition, and water content in the calf pre- and post-HD.

**Results:**

Mean UF volume was 8.13 ± 4.4 mL/kg/hr. Fluid removal was accompanied with effective mobilization of peripheral water (measured with MRI within the thigh) from 0.85 ± 0.21 g/mL to 0.83 ± 0.18 g/mL, and reduction in total body water (38.9 ± 9.4 L to 37.4 ± 8.6 L). However, directly-measured liver water content did not decrease (0.57 ± 0.1 mL/g to 0.79 ± 0.3 m L/g). Liver water content and IVC diameter were inversely proportional (*r* = − 0.57, *p* = 0.03), a relationship which persisted after dialysis.

**Conclusions:**

In contrast to the reduced total body water content, liver water content did not decrease post-HD, consistent with a diversion of blood to the hepatic circulation, in those with signs of greater circulatory stress. This novel observation suggests that there is a unique hepatic response to HD with UF and that the liver may play a more important role in intradialytic hypotension and fluid shifts than currently appreciated.

## Background

Determining the appropriate fluid balance is often challenging in hemodialysis (HD) patients. Fluid overload is common and is associated with increased risk of cardiovascular morbidity and mortality [1, 2]. By contrast, excessive fluid removal can lead to myocardial stunning, a reversible functional impairment of the myocardium due to ischemic injury which has been shown to be directly related to the degree of hemodynamic stress during dialysis [3]. Recurrent stress of this nature leads to progressive cardiac dysfunction as well as ischemic injury in other organs, including the brain [4, 5].

The liver is a central organ which receives a high proportion of cardiac output and holds a significant volume of blood (10–15% of total blood volume) [6]. It has a role in the maintenance of hemodynamic stability during circulatory stress by releasing some of this held blood to support the circulating volume [7]. It is also vulnerable to chronic hypervolemia, hypovolemia and endotoxemia, all of which are common in hemodialysis patients [8, 9]. Few studies have assessed the hepatic-splanchnic vascular system in end-stage renal failure, however, there has not been reported studies to evaluate the impact of fluid overload and ultrafiltration on liver water content [10–12]. Liver congestion can be quantified by a new magnetic resonance (MR) technique at our centre. Our aim with this present pilot study was therefore to determine the impact of hemodialysis upon hepatic congestion and explore the hepatic hemodynamic response to hemodialysis. Our objective was to assess the hepatic responses, which include the changes in liver water, liver stiffness and liver enzymes, to fluid removal during HD. We hypothesized that liver water would decrease during HD. In addition, we hypothesized that liver stiffness would decrease after HD.

## Methods

### Ethical approval

This observational study was approved by the Health Sciences Research Ethics Board at the University of Western Ontario (HSREB106868) and conducted according to the GCP/ICH guidelines and the Declaration of Helsinki.

### Study population

Patients from the prevalent chronic HD population cared for in the London Regional Renal Program (Ontario, Canada) were recruited to a cross-sectional study and written consent was obtained from all patients. For inclusion, patients had to have been receiving dialysis for at least 3 months. Exclusion criteria included the presence of liver disease or known risk-factors for liver disease (viral hepatitis, history of significant alcohol intake), contraindications to MR scanning (for the sub-study) and history of lower limb amputation. In addition, if the patients had any signs of inflammation, infection, maglignancy, and/or hemodynamic instability, they were excluded.

Baseline information was obtained for each subject, including age, gender, height, blood pressure, underlying renal disease, dialysis information (frequency, blood flow rate, dialysate flow rate, ultrafiltration volume, duration of dialysis session, and single-pool Kt/V urea measurements), and most recent residual renal function measurement.

Prior to and after their routine HD treatments, all subjects underwent clinical assessment of fluid status 30–60 min prior to their HD treatment. Multi-frequency bioimpedance analysis of total body water and magnetic resonance imaging (MRI) scans in supine position were also performed during this period. In addition, blood samples were taken to measure biomarkers such as hematocrit, creatinine, urea, bilirubin, liver enzymes, and sodium levels.

Bioimpedance analyzer were performed using the standard tetrapolar technique, with electrodes placed at both wrists and both ankles after subjects lay supine for at least 10 min (50 frequencies (5 to 1000 kHz), Body Composition Monitor®, Fresenius Medical Care Deutschland GmbH) [13].

**Fig. 1:**
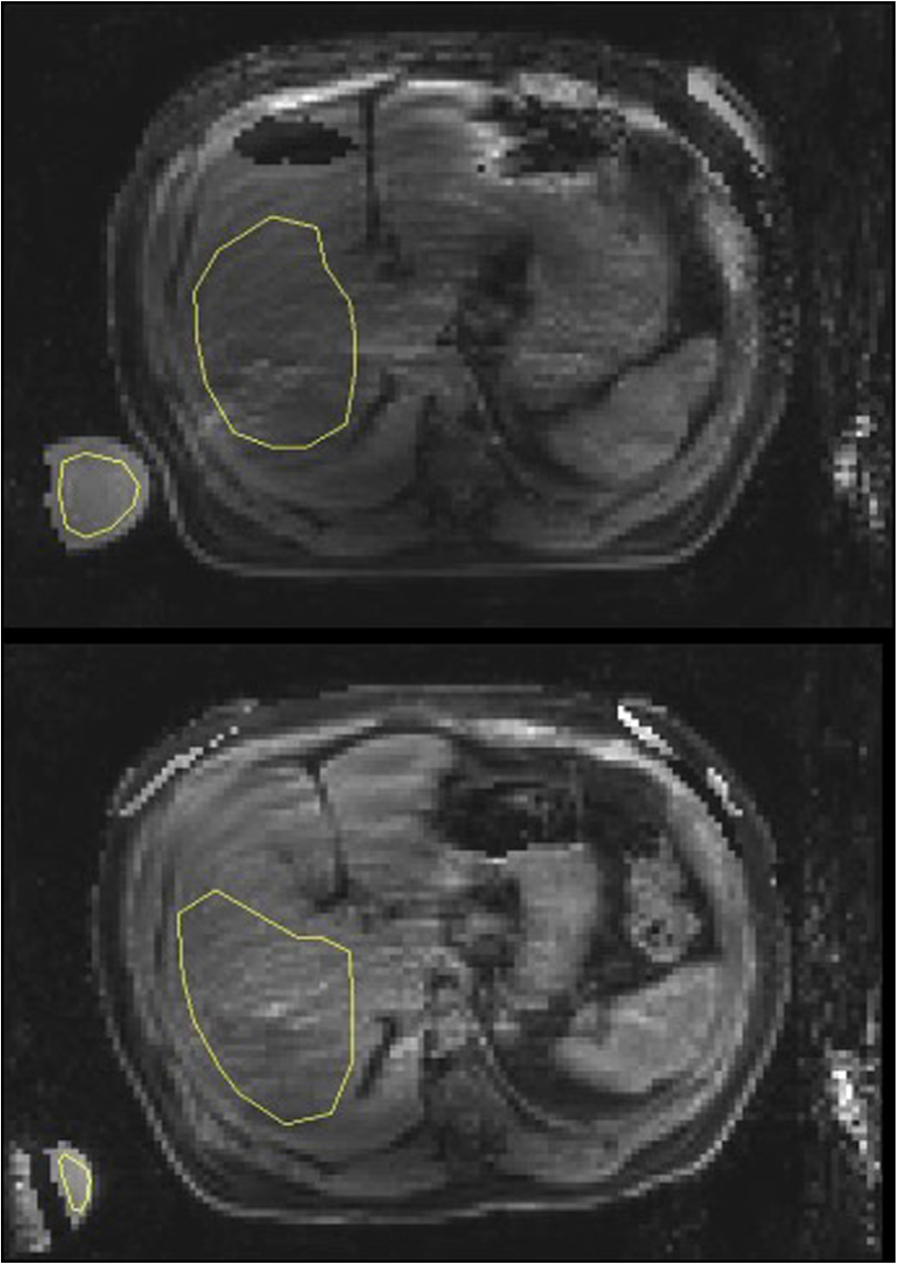
Water image from CSE-MRI reconstruction showing the Region of Interest (ROI) in the liver for which liver water content was computed. ROIs were drawn to avoid major vessels, and regions which contained water-fat swaps. A water phantom is shown in the lower left, which was used for normalization to obtain absolute. These two images were from Patient 14. Top: pre-dialysis (0.7) and bottom: post-dialysis (0.83)

Organ water content was quantified pre- and post- dialysis using 3D chemical shift encoded MRI (CSE-MRI) pulse sequence implemented on a 3 Tesla MRI scanner (MR 750, GE Healthcare, Waukesha, WI) [14]. This technique has been validated in clinical studies and employed in large randomized-controlled trials [15–17]. Imaging parameters were selected to cover the abdomen with proton density contrast: Field Of View (FOV) = 48x34x58 cm [3], matrix = 128x90x72, repetition time (TR) = 4.7 ms, flip angle = 3 deg. The acquisition was accelerated using ARC (autocalibration reconstruction for Cartesian imaging) in the phase and slice directions with a 32 channel Torso array for an overall acceleration factor of *R* = 3.9, permitting a complete volume to be acquired in a sub 20s breath hold. The images at the 6 echoes (echo time (TE) (ms) = [0.37 0.76 1.14 1.152 1.90 2.28]) were receive sensitivity corrected and processed to give water only and fat only images. Organ water content was then evaluated by drawing regions of interest (ROI) in each organ and normalizing by comparing with a water phantom that was included in the field of view [18]. For the hepatic ROI, we select the right lower lobe with 1 cm margin to exclude splanchnic vascular bed (Fig. 1). We also avoided the portal and hepatic vasculature. For the peripheral muscle water content, unaccelerated, high resolution, 3D CSE-MRI images (FOV = 48x38x48, matrix = 192x160x80) were also acquired of the thigh with otherwise similar parameters and processing, to quantify muscle water content. Finally, the infectior vena vaca (IVC) diameter was measured in an axial T2 weighted fast spin echo slice (FOV = 48 cm, thickness = 8 mm, matrix = 384 × 160, TE = 80 ms) approximately 5 cm below the diaphragm. Diameters were measured by two readers and averaged.

Measures of liver stiffness were obtained by a single, trained operator (CG) using transient elastography (Fibroscan® GE Healthcare) [19]. Measurements were obtained by the conventional clinical method, with the subject supine and 10 valid measurements obtained with the median value recorded. Unreliable results were deemed as those with an IQR of less than 33% of the median or in those in whom fewer than 10 valid results could be obtained.

All these measurements and assessments were repeated 30–60 min after their HD treatment.

### Statistical analysis

Data were analyzed using SPSS Version 22 (IBM, Chicago, US). All data were tested for normality using the Shapiro-Wilk test. Parametric data are described using the mean ± 1 standard deviation (SD) whereas non-parametric data were expressed using the median (interquatile range, IQR). Comparisons of continuous outcomes between two groups were performed using the independent t test for parametric data and Mann Whitney U test for non-parametric data. Comparisons of related outcomes at two different time points were performed using the paired t-test for parametric data and the Wilcoxon signed-rank test for non-parametric data. Bivariate correlation was assessed using Pearson’s correlation coefficient for parametric data and Spearman’s coefficient for non-parametric data. An alpha error of less than 5% (*p* < 0.05) was considered to be statistically significant.

## Results

### Study population

Eighteen patients consented to be part of the study from February 2015 to April 2016. Two subjects were excluded due to the presence of exclusion criteria. One subject was excluded from the analysis due to the diagnosis of metastatic cancer. Poor data quality meant that liver MRI data was not available for one subject. Fourteen subjects for whom liver MRI data was available formed the study population.

Characteristics of the study population are shown in Table 1. The majority of patients were male and had either diabetic or hypertensive nephropathy as the underlying etiology of their renal disease. Other etiologies included unspecified nephrotic syndrome (1) obstructive uropathy (1), lupus nephritis (1), renal dysplasia associaved with Prune-Belly syndrome (1) and IgA nephropathy (1). The majority of patients (86%) were dialyzed via central catheter access with the remainder via peripheral fistula access. Echocardiographic of all these patients showed normal ejection fraction (EF) > 55–60% except one patient who had EF 35–40%.

**Table 1:** Study Population Characteristics

Age (yrs)	58.6 ± 18
Gender (M/F)	13/1
BMI (g/m^2^)	23.8 ± 2.6
Diabetes (Y/N)	5/9
Known CAD (Y/N)	5/9
Aetiology of ESRF (DM/HTN/Other)	5/3/6
ACEi/ARB (Y/N)	5/9
Calcium channel blocker (Y/N)	10/4
Beta blocker (Y/N)	10/4
Alpha blocker (Y/N)	2/12
Diuretic (Y/N)	6/8
Use of ESA (Y/N)	10/4
Time on dialysis (months)	9 (4–172)
Time receiving HD (months)	6 (1–172)
Previous transplant (Y/N)	4/10
Current HD access (CVC/AVF)	12/2
Hemoglobin (g/L)	107 ± 13
Haematocrit (L/L)	0.324 ± 0.04
Sodium (mmol/L)	136 ± 3.9
Albumin (g/L)	38.5 ± 6.2
Bilirubin (μmol/L)	4.19 ± 1.2

*M* male, *F* female, *Y* yes, *N* no, *CAD* coronary artery disease, *ESRF* end-stage renal failure, *HTN* hypertension, *DM* diabetes, *ACEi* angiotensin converting enzyme inhibitor, *ARB* angiotensin receptor blocker, *ESA* erythopoeisis-stimulating agent, *CVC* central venous catheter, *AVF* arteriovenous fistula. All blood results represent pre-dialysis values

All subjects underwent their prescribed length of dialysis session as part of the study. Ultrafiltration volumes ranged from 0 to 4090 mL (mean 8.13 ± 4.4 mL/kg/hr). Two patients experienced symptomatic intra-dialytic hypotension (IDH), with dizziness or severe cramps. Three subjects had IDH defined as a nadir systolic blood pressure of less than 100 mmHg, whilst six subjects experienced a fall in systolic blood pressure of at least 40 mmHg during dialysis.

### Measures of hepatic water and central volume

**Fig. 2:**
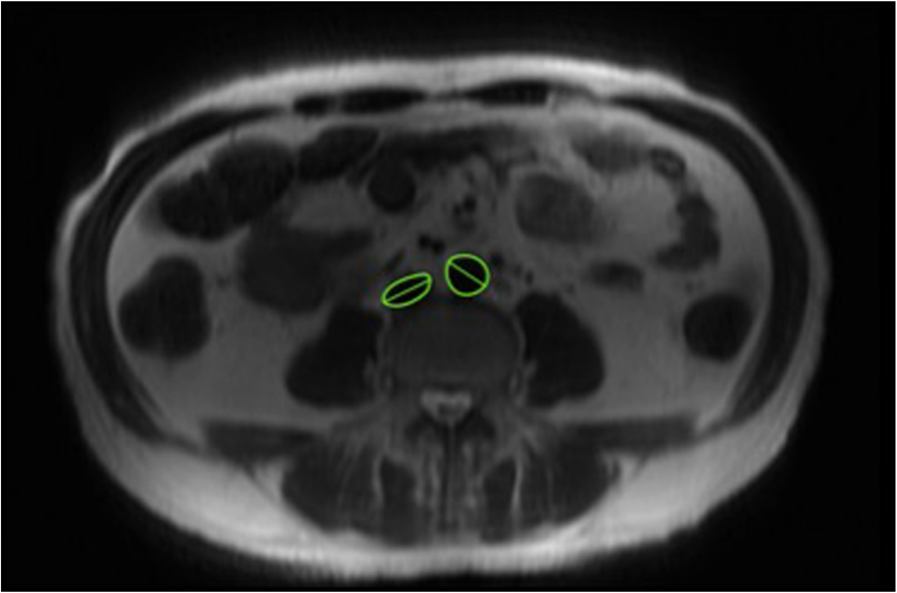
FSE image indicating the inferior vena cava and abdominal aorta, and the major diameter of each

Mean pre-dialysis hepatic water content was 0.63 ± 0.19 g/mL (range 0.34 g/mL to 1.05 g/mL). There was no association between hepatic water and clinically or bioimpedance-derived measures of hypervolemia. There was also no significant relationship between liver stiffness and hepatic water content (*r* = − 0.02, *p* = 0.93; Table 2). Higher hepatic water content was, however, associated with a smaller IVC diameter (*r* = − 0.57, *p* = 0.03) (Fig. 2) and with a higher pre-dialysis hematocrit (*r* = 0.61, *p* = 0.048).

**Table 2:** The relationship between ultrafiltration, liver stiffness and liver water content

Patient Number	Ultrafiltration volume (mL)	% change in MRI water	% change in liver stiffness
1	0	80%	27%
2	200	−5%	12%
3	1200	26%	−17%
4	1400	−52%	25%
5	1600	21%	−10%
6	1800	126%	115%
7	2100	−30%	5%
8	2340	22%	33%
9	2400	0%	50%
10	2800	−3%	26%
11	2900	−21%	0%
12	3500	32%	−20%
13	3600	37%	9%
14	4090	9%	−59%

**Fig. 3:**
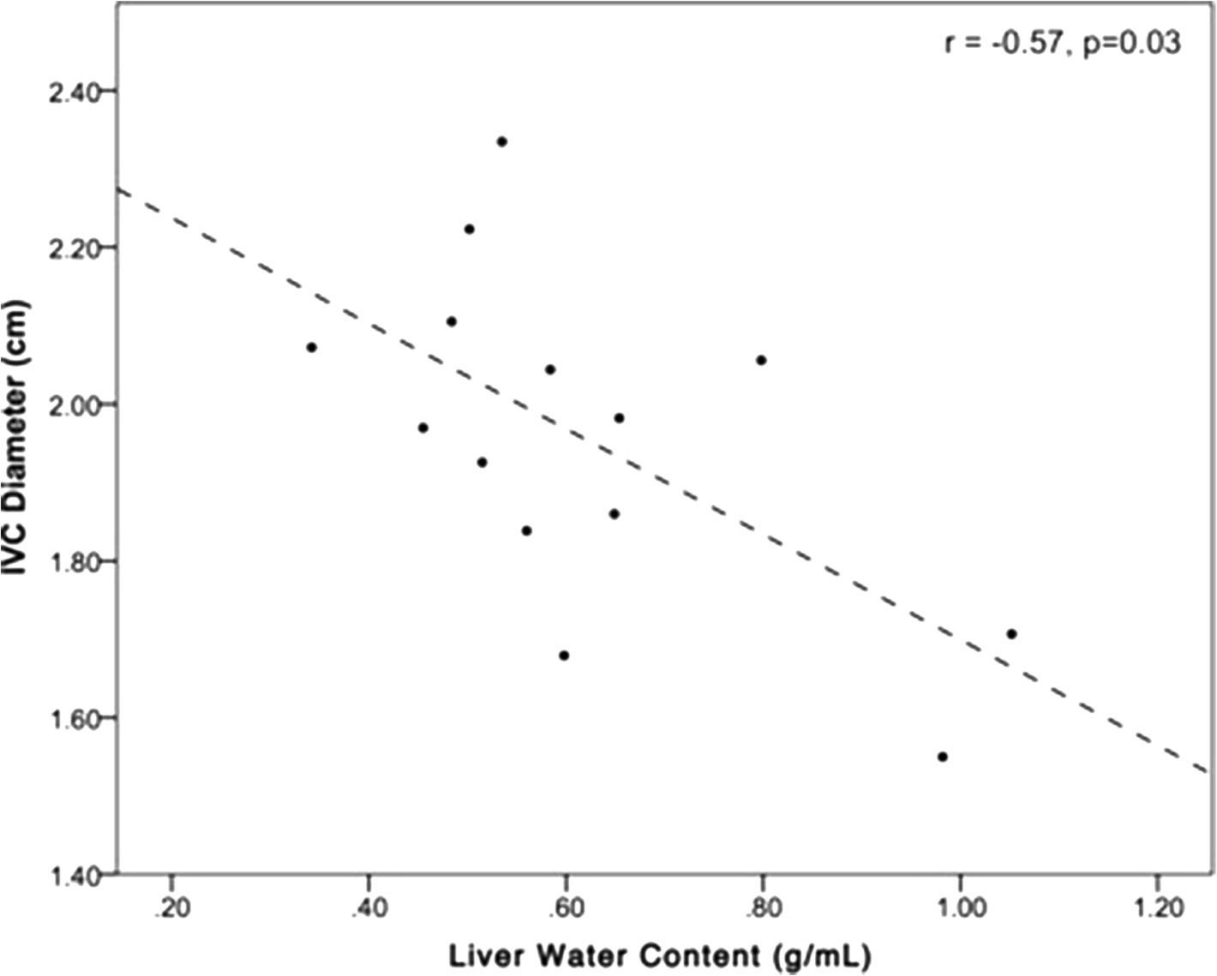
Relationship between liver water content and IVC diameter pre-dialysis

**Fig. 4:**
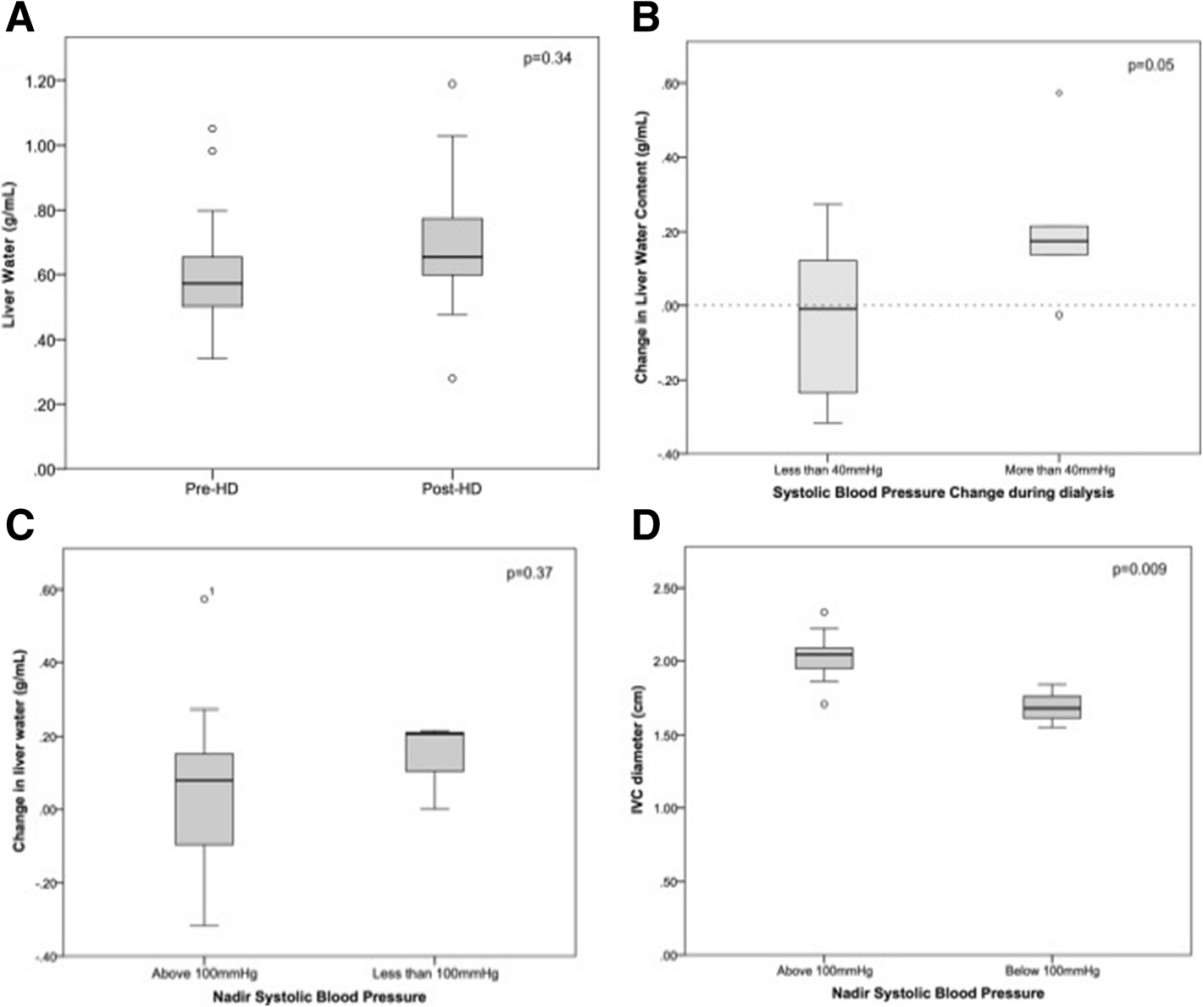
Change in liver water content with dialysis and the relationship with haemodynamic stability during HD. **a**: Liver water before and after dialysis. **b**: Liver water and fall in blood pressure **c**: Liver water and nadir blood pressure. **d**: IVC diameter and nadir blood pressure

The mean liver stiffness values were 5.0 ± 2.07 kPa and 6.3 ± 2.31 kPa pre and post-hemodialysis (*p* = 0.20). There was no statistically significant change in the mean liver water content after HD (from 0.63 ± 0.2 g/mL to 0.70 ± 0.2 g/mL, *p* = 0.34) (Fig. 3). The liver water content increased in eight subjects but fell in the remaining six subjects. The magnitude of the change in liver water content pre- and post- hemodialysis was not associated with ultrafiltration volume (*r* = − 0.04, *p* = 0.88) (Fig. 4) or the change in liver stiffness values (*r* = 0.06, *p* = 0.84). There were no clear clinical factors which were significantly associated with the direction of change in liver water content, including age, co-morbidity or fluid status before dialysis. However, liver water content rose in all patients with intra-dialytic hypotension, which was defined as > 40 mmHg drop in blood pressure with/without symptoms and/or a nadir blood pressure < 100mHg based on previous studies [20]. Furthermore, there was a greater increase in liver water content in those whose systolic blood pressure fell by more than 40 mmHg during dialysis (0.21 ± 0.2 g/mL vs. -0.04 ± 0.2 g/mL, *p* = 0.05) (Fig. 3).

IVC diameter did not change significantly after dialysis. Nevertheless, the correlation between liver water content and IVC diameter persisted (*r* = − 0.60, *p* = 0.03). Pre-dialysis IVC diameter was lower in those who suffered IDH, with a nadir blood pressure < 100mHg, (2.02 ± 0.17 cm vs 1.69 ± 0.14 cm, *p* = 0.009).

### Peripheral and Total body water content

Pre-dialysis, total body water was 39.0 ± 9.4 L with intra-dialytic weight gains of between − 3 and 5.9 kg with median weight gain of 1.65 kg. One subject was 3 kg under their target weight prior to dialysis and bioimpedance measured that he was 1.2 L below his ideal fluid status. Full bioimpedance analysis was unobtainable in the one subject who demonstrated clinically significant fluid overload, being almost 6 kg over the target weight with profound peripheral edema. Pre-dialysis MRI-measured limb (thigh) water content was associated with a higher total body water (*r* = 0.59, *p* = 0.06), higher intracellular water volume (*r* = 0.64, *p* = 0.02) and a higher diastolic blood pressure (*r* = 0.70, p = 0.02). After dialysis, total body water fell by a mean of 1.59 L to 37.4 ± 8.4 L and thigh water content decreased from 0.85 ± 0.21 g/mL to 0.83 ± 0.18 g/mL, although this was not statistically significant (*p* = 0.55).

### Change in hematocrit and liver biochemistry

The liver enzymes were measured pre- and post-dialysis. There were significant changes in aspartate aminotransferase, alkaline phosphatase, hematocrit and total bilirubin values. The alanine aminotransferase and gamma-glutamyl transferase values were not significantly altered. After using the correction factor based on the published data by Schneditz et al., however, we still found statistically significant changes in some of the liver enzymes, including aspartate transaminase, alkaline phosphatase and gamma-glutamyl transpeptidase [21]. Please see Table 3. However, these values did not correlate with the changes in liver water.

**Table 3:** The changes in the hematocrit, bilirubin and liver enzymes values

	Pre-dialysis	Post-dialysis	*P*-values
Aspartate aminotransferase, U/L	16 ± 3.8	21 ± 11.9	< 0.01
Alanine aminotransferase, U/L	12 ± 5.8	19 ± 17.8	0.58
Gamma-glutamyl transferase, U/L	23 ± 4.2	23 ± 5.7	0.95
Alkaline phosphatase, U/L	105 ± 47.1	111 ± 40.6	< 0.01
Total bilirubin, umol/L	4.19 ± 1.17	5.00 ± 1.56	< 0.01
Hematocrit, L/L	0.32 ± 0.04	0.35 ± 0.04	< 0.01

## Discussion

We are unaware of other studies that specifically compare the effects of HD with ultrafiltration related to liver water content with peripheral and total body water. Among the fourteen subjects, there was marked variation in liver water content pre-dialysis, surprisingly with a lower value than expected compared to non-dialysis subjects in other studies [22], even in those with fluid overload. It has been shown that central volume is relatively preserved with ultrafiltration but organ perfusion is vulnerable to smaller shifts in hemodynamic stability [23, 24]. In our study, whilst MR-measured peripheral water content and commonly-used markers of overall fluid status tended to correlate well with each other, there was no demonstrable relationship between hepatic water content and the majority of volume status markers.

Liver water was measured using an MR technique initially designed to assess hepatic steatosis, by separating it from water content. This technique has been validated in clinical studies and employed in large randomized-controlled trials [15–17]. The value reported represents water concentration. In the liver, as there is no significant extravascular, extracellular space, this is a composite of intracellular fluid, bile and largely intravascular volume. With regards to MR imaging, we selected as large a region of interest as possible at several different imaging slices, excluding major vessels. Therefore, the changes in the liver water content was unlikely due to imaging selection bias. There have been several studies that assessed the splanchnic perfusion during hemodialysis and they found that there were increased hepato-splanchnic vasoconstriction with ultrafiltration [10–12]. However, these studies only evaluated the splanchnic vascular system. We are the first to report the effect of fluid removal during hemodialysis on intrahepatic liver water content. Our paradoxical result, a non-significant increase in liver water content, suggested that liver perfusion is a complicated system and cannot be explained by changing in one vascular system, i.e. the splanchnic vasculature. In addition, a recent pilot study showed an increased in hepatic flow while a reduction in portal flow during dialysis [25]. It may involve the hemodynamic changes in the renal-gastro-hepatic axis with end stage renal disease, in addition to amount of uremic solutes flow through the hepatic system.

In health, in response to a reduction in circulating volume, there is immediate contraction of liver volume and expulsion of pooled blood. In this study, not only did we observe that liver water content reduced to a lesser extent than peripheral fluid, actually in many patients it increased. The changes in liver water content in our subjects were larger than expected by the confidence interval alone. Our observations suggest this physiological reaction does not occur in all patients undergoing HD. This response is not associated with comorbidities, drugs (such as antiplatelet, antihypertensive and erythropoietin agents) and blood hematocrit, although our study may be underpowered to really study this. This may be due to differing levels of circulatory stress endured, with some subjects not meeting the requirements for activation of hepatic venoconstriction [25]. Another explanation is that some subjects exhibited an abnormal hepatic response to the circulatory stress of dialysis, with a diversion of blood flow into the hepatic circulation. This is supported by the observation that liver water concentration increased significantly more in patients who sustained a considerable fall in their blood pressure during HD. The negative impacts of ultrafiltration on myocardial perfusion have been well described [26, 27]. The presence of both cardiac and baseline autonomic dysfunction is also variable in HD patients and this may additionally explain the heterogeneity observed [28, 29].

Both passive distension and active venodilatation occur in the face of hypervolaemia to accommodate additional circulatory volume. Chronic congestion may lead to increased hepatic size, but also leads to hepatic injury and fibrosis, limiting the distensibility of liver tissue. It is unclear whether patients with end-stage renal failure undergoing regular dialysis develop chronic hepatic congestion. In the majority of subjects in this study, liver stiffness was within the normal range, making significant fibrosis unlikely. The liver enzymes were all within target range. The changes in liver enzymes, hematocrit and bilirubin before and after dialysis did not correlate with the changes in liver water. The changes in these values could be due to hemoconcentration post ultrafiltration.

Water concentration prior to HD was nevertheless highly variable but with no clear relationship to MR-measured, bioimpedance-derived or clinical assessments of fluid status. Furthermore, all patients except one had normal cardiac function with no significant valvular disease. Therefore, cardiac dysfunction could not explain our findings. One subject clinically appeared to be significantly fluid-overloaded, yet their liver water concentration was not elevated compared to other subjects. By contrast, their peripheral water content measured by MRI followed the appropriate responses to ultrafiltration. Such results suggest that liver water concentration does not accurately reflect fluid status in dialysis patients. In addition, liver water concentration was inversely associated with one marker of central volume status: IVC diameter. IVC size reflects central venous pressure and is used as a marker of fluid status. A higher IVC diameter is reflective of circulatory volume overload, therefore one would expect the relationship with liver water concentration to be the inverse of what was observed.

Although the MRI technique has been used in other non-dialysis studies, this pilot study was designed to explore the effects of HD upon liver congestion and, as such, was not powered to conclusively establish trends, therefore these results must be confirmed in larger studies. Furthermore, the MRI technique is associated with a measurement error of 0.1 g/mL. As such, a value of 1.0 g/mL may actually reflect 0.9 g/mL. This may explain the extreme values of liver water content. In addition, to capture accurate measurements, breathing holding technique was used, which could also be a source of error if the patients could not hold their breath for 20 s. These are several limitations to consider which would be useful to address when designing future studies. The relatively small number of patients limited the statistical power to do further subgroup analyses. However, it is not uncommon for this type of translational research [10, 12]. We did not measure cardiac function during the hemodialysis as part of the study; however, static measures of cardiac function did not show any significant cardiac dysfunction prior to our study in these patients. Finally, subjects were not fast for a specific time prior to and during dialysis; ingestion of a large or lipid-rich meal in the few hours prior to and during dialysis my have affected individual results, as demonstrated by more recent studies [30]. We did not assess other biomarkers, such as vasopressin or copeptin, that may play a role in hepato-splanchnic circulation in this population. Further assessments of these biomarkers in the future study should be considered.

## Conclusions

In conclusion, we have shown that liver water content does not reflect volume status in the hemodialysis patient, nor does it reduce with ultrafiltration, unlike peripherally-held volume. Instead, there appears to be a complex relationship between liver water and hemodynamic stability during HD, suggesting that the hepatic circulatory system plays a role in the evolution of circulatory stress. Understanding the physiology underlying this novel observation may help to explain the hemodynamic response to HD.

## Abbreviations

ARC: Autocalibration reconstruction for cartesian imaging
CSE-MRI: Chemical shift encoded magnetic resonance imaging
EF: Ejection fraction
FOV: Field of view
HD: Hemodialysis
IDH: Intra-dialysis hypotension
IQR: Interquatile range
IVC: Infectior vena vaca
MRI: Magnetic resonance imaging
ROI: Regions of interest
SD: Standard deviation
